# Exercise and Redox Status Responses Following Alpha-Lipoic Acid Supplementation in G6PD Deficient Individuals

**DOI:** 10.3390/antiox7110162

**Published:** 2018-11-12

**Authors:** Kalliopi Georgakouli, Ioannis G. Fatouros, Apostolos Fragkos, Theofanis Tzatzakis, Chariklia K. Deli, Konstantinos Papanikolaou, Yiannis Koutedakis, Athanasios Z. Jamurtas

**Affiliations:** 1 School of Physical Education and Sport Science, University of Thessaly, Karies, 42100 Trikala, Greece; kgeorgakouli@gmail.com (K.G.); fatouros@otenet.gr (I.G.F.); apostolisfragkos@yahoo.gr (A.F.); tzatzakis23@gmail.com (T.T.); delixar@pe.uth.gr (C.K.D.); guspapa93@gmail.com (K.P.); y.koutedakis@pe.uth.gr (Y.K.); 2 Center for Research and Technology Hellas (CERTH) at Thessaloniki, Institute for Research and Technology of Thessaly (I.RE.TE.TH) at Karies, 42100 Trikala, Greece; 3 Faculty of Arts, University of Wolverhampton, WS1 3BD Walshall, UK

**Keywords:** antioxidant, enzymopathy, oxidative stress, exercise performance, muscle

## Abstract

G6PD deficiency renders cells more susceptible to oxidative insults, while antioxidant dietary supplementation could restore redox balance and ameliorate exercise-induced oxidative stress. To examine the effects of alpha-lipoic acid (ALA) supplementation on redox status indices in G6PD deficient individuals, eight male adults with G6PD deficiency (D) participated in this randomized double-blind placebo-controlled crossover trial. Participants were randomly assigned to receive ALA (600 mg/day) or placebo for 4 weeks separated by a 4-week washout period. Before and at the end of each treatment period, participants exercised following an exhaustive treadmill exercise protocol. Blood samples were obtained before (at rest), immediately after and 1h after exercise for later analysis of total antioxidant capacity (TAC), uric acid, bilirubin, thiobarbituric acid reactive substances (TBARS) and protein carbonyls (PC). ALA resulted in significantly increased resting TAC and bilirubin concentrations. Moreover, TAC increased immediately and 1h after exercise following both treatment periods, whereas bilirubin increased immediately after and 1h after exercise following only ALA. No significant change in uric acid, TBARS or PC was observed at any time point. ALA supplementation for 4 weeks may enhance antioxidant status in G6PD individuals; however, it does not affect redox responses to acute exercise until exhaustion or exercise performance.

## 1. Introduction

Glucose-6-phosphate dehydrogenase (G6PD) enzyme deficiency is the most common enzymopathy, affecting more than 400 million people worldwide [1]. G6PD is involved in the generation of NADPH, a reducing agent that participates in various anabolic pathways and the regeneration of reduced glutathione (GSH) [2]. G6PD deficient cells have lower concentrations of GSH. Since GSH protects cells against reactive oxygen species (ROS), G6PD deficiency renders cells susceptible to oxidative stress and its consequences.

Deficient G6PD activity is well examined in red blood cells (RBC), as it often results in clinical manifestation of mild to severe haemolytic anaemia following exposure to various oxidative agents such as certain drugs [1]. Although G6PD deficient activity is also present in other cells such as white blood cells and myocytes, theoretically causing different clinical symptoms under conditions of increased oxidative stress, research in this field is limited.

Intense exercise results in an increased use of oxygen by the working muscles and generation of ROS [3]. The antioxidant defence systems minimize the damage caused by ROS; however, when these systems cannot efficiently remove the ROS, an imbalance in favour of ROS production occurs which is known as oxidative stress. Since G6PD deficient individuals have lower endogenous antioxidant system, intense exercise could result in increased oxidative stress in RBC and myocytes, which in turn could cause haemolytic anaemia and/or muscular symptoms, respectively.

For this reason, it has been postulated that G6PD-deficient individuals should avoid performing heavy physical exercise [4]. Indeed, symptoms such as haemolysis, muscle degeneration, myalgia and myoglobinuria have been reported following intense exercise [5,6,7,8]. Nevertheless, experimental studies have not detected greater levels of exercise-induced oxidative stress or signs of haemolysis in G6PD deficient individuals compared to individuals with normal G6PD activity [9,10,11]. On the other hand, it is well known that physical activity/exercise contributes to the maintenance and promotion of health. To date there are no guidelines for exercise in G6PD deficient individuals.

Enhancement of antioxidant defence mechanisms in G6PD-deficient individuals could result in safer participation in exercise. Theoretically, restoration of GSH concentrations would enhance these mechanisms, rending cells less susceptible to oxidative insults. Since GSH formation greatly depends on cysteine, dietary supplements with antioxidants or cysteine donors such as α-lipoic acid (ALA) could positively influence recovery following exercise in G6PD deficient individuals. Trace amounts of LA are synthesized by the human body, while exogenously supplied ALA is reduced to dihydrolipoate (DHLA) by NADH- or NADPH-dependent enzymes in several tissues [12,13,14]. DHLA provides cysteine by reducing cystine, which is abundant in the extracellular compartment [15] and it is then oxidized to ALA. ALA has the ability to continuously supply cysteine as long as it gets reduced to DHLA by intracellular enzymes [15,16]. Cysteine is a precursor for glutathione synthesis, and its intracellular availability plays a major role in cellular glutathione levels [16]. Since ALA has the ability to continuously supply cysteine, it increases intracellular GSH. Indeed, studies have shown that ALA supplementation increases intracellular GSH levels in various cell types and tissues [15,17,18] in various pathological conditions [18] including G6PD deficiency [19]. Thus ALA supplementation could be beneficial for individuals with G6PD deficiency who perform exercise. The purpose of this study was to examine the effects of 4-week ALA supplementation on blood redox status in males with G6PD deficiency following acute exercise.

## 2. Materials and Methods

### 2.1. Subjects

Eight healthy males (age: 38.4 ± 5.6 years) with G6PD deficiency participated in this study. Participants were recreationally trained, as evidenced by their maximal oxygen consumption (VO_2_max) level (mean VO_2_max: 40.7 ± 1.7 mL/kg/min) and were non-smokers. Participants abstained from any vigorous physical activity and the consumption of caffeine and alcohol for at least two days before each exercise trial.

Exclusion criteria included intake of performance-enhancing or antioxidant supplements and medications over the last 6 months and during the study, a known ALA intolerance, and any medical condition that contraindicates participation to exercise.

Participants were informed about the study procedures, risks and benefits involved in the study and signed an informed consent form. Procedures were in accordance with the 1975 Declaration of Helsinki (2000). Approval was previously obtained from the Institutional Review Board of the University of Thessaly (protocol number 1076). Moreover, this study was registered at ClinicalTrials.gov as NCT02937363 [20].

### 2.2. Experimental Design

The study was a randomized double-blind placebo-controlled crossover trial. Participants were randomly assigned to receive ALA (600 mg/day) or placebo (PL) for 4 weeks separated by a 4-week washout period. Before and at the end of each period, participants exercised on a treadmill (a total of four exercise trials) at an intensity corresponding to 70–75% of their VO_2_max for 45 min and then at 90% of their VO_2_max until exhaustion. Blood samples were obtained before (at rest), immediately after and 1 h after exercise.

Participants recorded their diet for two days before their first exercise trial and they followed the same diet before each of the subsequent exercise trial. Moreover, participants were instructed to avoid any strenuous physical activity for at least two days before each exercise trial. Exercise trials were performed in the morning after an overnight fast (10 h).

### 2.3. Physical Characteristics and Physical Activity Level

Body height was measured to the nearest 0.1 cm and body weight to the nearest 0.1 kg (Beam Balance, Seca, UK), with the participants lightly dressed and barefoot. Body fat percentage was estimated by dual-energy X-ray absorptiometry (DEXA) (Lunar DPX NT, GE Healthcare, UK).

The level of physical activity of the participants was determined by using the Greek version of the International Physical Activity Questionnaire (IPAQ) [21].

### 2.4. Exercise Performance

*Aerobic fitness* (*VO_2_max test*): VO_2_max was determined using a treadmill test to exhaustion. The test commenced at a speed corresponding to ~60% of each participant’s predicted VO_2_max (Karvonen Formula: Target heart rate = ((220 − age in years) − resting heart rate) × (% intensity) + resting heart rate) for the first two minutes. Then, speed increased by 0.5 km/h every minute until exhaustion. Heart rate was monitored every minute (Polar RC3 GPS HR; Polar Electro Oy, Kempele, Finland). VO_2_ uptake, and CO_2_ production were continuously recorded via a computerized online gas analysis system (SensorMedics 2900c, SensorMedics Corporation, Yorba Linda, CA, USA) calibrated to known gases.

*Aerobic endurance* (*time to exhaustion test*): Time to exhaustion was measured using an exhaustive treadmill exercise protocol that has been previously described [9].

*Muscle function and performance* (*isometric peak torque*): An isokinetic dynamometer (Cybex, HUMAC NORM 360, Ronkonkoma, NY, USA) was used for the evaluation of knee extensor performance of both legs by measuring isometric peak torque at 90° knee flexion before each exercise trial. The best of the three maximal voluntary contractions (MVC) (performed with one minute rest between efforts) was recorded. If the difference between the lower and the higher torque values exceeded 10% the measurements were repeated.

### 2.5. Blood Collection and Handling

To determine whether participants were G6PD deficient before their enrolment to the study, a blood sample was obtained for the measurement of G6PD activity in red blood cells. A portion of whole blood was collected and handled as previously described [19].

Blood samples during the study were obtained for later analysis of total antioxidant capacity (TAC), uric acid, bilirubin, thiobarbituric acid reactive substances (TBARS) and protein carbonyls in serum. Blood handling for serum preparation has been described elsewhere [19].

### 2.6. Blood Sample Analysis

All indices were analysed in duplicates. G6PD activity was measured on the day of blood sample collection. Indices of blood redox status were measured after samples had undergone only one freeze-thaw cycle.

For TAC determination 20 μL of serum were added to 480 μL of 10 mM sodium potassium phosphate (pH 7.4) and 500 μL of 0.1 mM 2,2-diphenyl-1-picrylhydrazyl (DPPH) free radical, the samples were incubated in the dark for 30 min at room temperature and then they were centrifuged for 3 min at 20,000× *g*. Their absorbance was read at 520 nm.

Uric acid and bilirubin in serum were determined using commercially available kits (Zafiropoulos Diagnostica, Athens, Greece) in a Clinical Chemistry Analyzer Z 1145 (Zafiropoulos Diagnostica, Athens, Greece). Several studies have shown that bilirubin has antioxidant activities [22] that contribute towards the total antioxidant capacity whereas reports indicate that uric acid contributes significantly towards the plasma total antioxidant activity [23]. Therefore, these two indices were additionally included in the biochemical analyses to elucidate the role of ALA supplementation on exercise-induced oxidative stress.

For TBARS determination 100 μL of serum was mixed with 500 μL of 35% TCA and 500 μL of Tris–HCl (200 mM, pH 7.4). The samples were incubated for 10 min at room temperature, then 1 mL of 2 M Na2SO4 and 55 mM thiobarbituric acid solution were added, and the samples were incubated at 95 °C for 45 min. After 5 min on ice, 1 mL of 70% TCA was added and the samples were vortexed, then centrifuged at 15,000× *g* for 3 min, and the absorbance of the supernatant was read at 530 nm. A blank was also run along with samples during the measurement. Calculation of TBARS levels was based on the molar extinction coefficient of malondialdehyde.

For the determination of protein carbonyls 50 μL of 20% TCA were added to 50 μL of serum. The samples were incubated in an ice bath for 15 min and centrifuged at 15,000× *g* for 5 min at 4 °C. The supernatant was discarded, and 500 μL of 10 mM 2,4-dinitrophenylhydrazine (in 2.5 N HCL) for the samples or 500 μL of 2.5 N HCL for the blanks were added. All samples were incubated in the dark at room temperature for 1 h, with intermittent vortexing every 15 min, and were then centrifuged at 15,000× *g* for 5 min at 4 °C. The supernatant was discarded, 1 mL of 10% TCA was added, vortexed, and centrifuged at 15,000× *g* for 5 min at 4 °C. The supernatant was discarded, 1 mL of ethanol-ethyl acetate was added, vortexed, and centrifuged at 15,000× *g* for 5 min at 4 °C. This step was repeated twice and then the supernatant was discarded, 1 mL of 5 M urea (pH 2.3) was added, vortexed, and incubated at 37 °C for 15 min. They were centrifuged at 15,000× *g* for 3 min at 4 °C, and the absorbance was read at 375 nm. Calculation of protein carbonyl concentration was based on the molar extinction coefficient of dinitrophenylhydrazine.

### 2.7. Statistical Analysis

Power analysis performed with data from a previous [19] showed that the minimum required sample size was 8 for a 2-sided type 1 error of 5%.

Data are expressed as mean ± SEΜ. Changes in dependent variables were examined with a two-way repeated-measures ANOVA [condition (ALA and PL) × time (before exercise, immediately after exercise, one hour after exercise)]. If a significant interaction was detected, pairwise comparisons were performed through simple contrasts and simple main effects analysis using the Bonferroni test method. Pre- and post-measurements within conditions were compared using paired t-tests. Significance was set at *p* ≤ 0.05. The statistical programme used was SPSS version 18.0 (SPSS Inc., Chicago, IL, USA).

## 3. Results

All participants completed both arms of the study, while no adverse effect was reported. Erythrocyte G6PD activity was 0.502 ± 0.01 U/g Hb (with normal ranging from 4.5 to 13.5 U/g Hb).

Baseline physical characteristics of the participants were similar between ALA and PL (Table 1), indicating that measurements were performed under the same conditions. Physical characteristics did not change throughout the study, except for resting heart rate (HR), which decreased following ALA (Table 1). Regarding exercise performance, treadmill time to exhaustion and MVC did not change throughout the study (Table 2).

**Figure 1:**
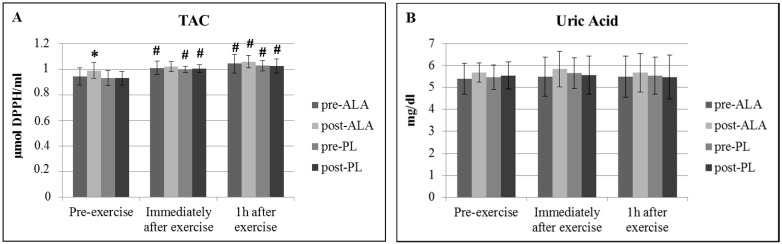

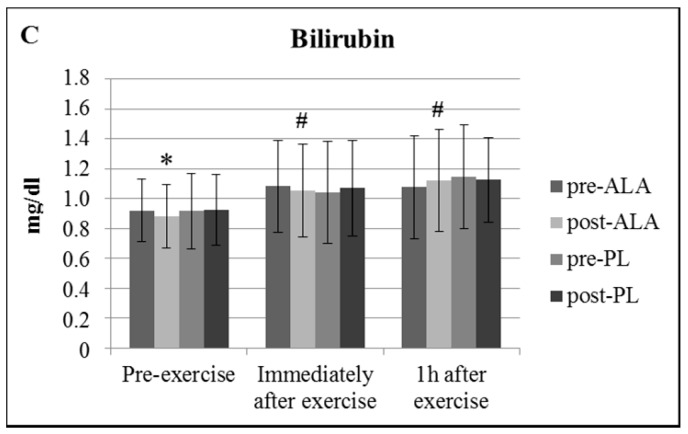
Mean (±SEM) changes in indices of antioxidant status ((**A**): TAC; (**B**): Uric Acid; (**C**): Bilirubin)). * Significant difference from pre-ALA at the same condition; **^#^** Significant difference from pre-exercise; TAC: Total Antioxidant Capacity.

**Figure 2:**
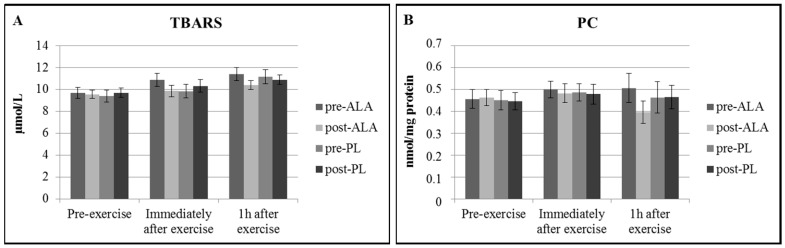
Mean (±SEM) changes in indices of oxidation ((**A**): TBARS; (**B**): PC). TBARS: Thiobarbituric acid reactive substances; PC: Protein Carbonyls.

Regarding the indices of blood redox status, there was a significant increase in resting TAC (Figure 1A) and a significant decrease in resting bilirubin (Figure 1C) concentrations following ALA. Moreover, TAC increased immediately after and one hour after exercise following both ALA and PL (Figure 1A), whereas bilirubin increased immediately after and one hour after exercise only following ALA (Figure 1B). Moreover, there was a non-significant (*p* = 0.059) increase in bilirubin one hour after exercise following PL (Figure 1C), and a non-significant (*p* = 0.06) increase in uric acid immediately after exercise following ALA (Figure 1B). TBARS did not significantly change at any time point in both conditions (Figure 2A).

## 4. Discussion

The purpose of the present study was to investigate the effect of ALA supplementation on indices of blood redox status in males with G6PD deficiency. We hypothesized that antioxidant supplementation would improve endogenous antioxidant status and responses to exercise, rending these individuals less susceptible to exercise-induced oxidative stress and improving exercise performance. The results showed that ALA supplementation for 4 weeks may contribute to enhanced antioxidant status; however, responses to acute exercise until exhaustion and exercise performance do not change.

Indices of redox status in serum usually reflect the overall redox status of the body; therefore, changes in these indices could provide information on the effect of ALA supplementation on the working muscles. TAC, as its name indicates, is a measurement of the cumulative action of all the antioxidants present in body fluids (including serum), providing an integrated parameter of antioxidants [24]. ALA supplementation resulted in a significant increase in resting TAC. A previous study from our laboratory with ALA supplementation had a similar effect in both deficient and non-deficient physically active individuals with G6PD deficiency [19]. In addition, TAC increased immediately after and one hour after exercise in all treatment periods (before and after ALA and placebo treatment). Increasing TAC levels in response to exercise to exhaustion has been previously reported [9] suggesting that acute exercise activates antioxidant defence mechanisms. Thus TAC in G6PD deficient individuals may not differ from those in non-deficient ones [9] and, even though ALA may further enhance TAC, no clear benefit of antioxidant supplementation can be observed following exercise.

No significant change in uric acid both at rest and following exercise was found. Nevertheless, a non-statistically significant (*p* = 0.06) increase immediately after exercise following ALA supplementation was observed. Previous data reported that ALA supplementation resulted in significantly increased uric acid in individuals with G6PD deficiency but not in those with normal enzyme activity [19]. Uric acid is main component of serum TAC [25]. However, a positive relationship between uric acid and TAC that has been previously described [26] was not evident in the present study. It is likely that uric acid levels were also affected by other factors than redox status. Another hypothesis is that other TAC components than uric acid (i.e., bilirubin, vitamin C) may have contributed to increased TAC levels.

Bilirubin increased significantly following ALA supplementation as a response to exercise (immediately after and 1 h after). As already mentioned, bilirubin is a component of TAC, and treatment with ALA supplementation influenced concomitant changes in these indices. In addition, bilirubin is also an indicator of erythrocytes lysis. It is known that non-muscle damaging exercise leads to short-lived haemolysis, which is most likely attributable to the break-up of erythrocytes due to impact forces during exercise [27]. Theoretically, since G6PD deficient erythrocytes may be more susceptible to oxidative stress, acute exercise could lead to haemolysis; however, no sign of haemolysis was observed. This is in accordance with a previous study that used a similar exercise protocol [9]. Nonetheless, in another study where G6PD deficient participants performed high-intensity eccentric exercise [11], marked and long-lived increases in bilirubin and haemoglobin were observed, indicating haemolysis up to 4 days following exercise. It is noteworthy that the levels of these indices peaked between 2 and 3 days, and not immediately after exercise. In the present study, blood samples were obtained only immediately after and 1 h after exercise, and that does not allow us to draw safe conclusions on whether the exercise protocol used cause haemolysis.

Resting levels of lipid peroxidation as examined by measuring TBARS concentration in serum did not change at any treatment period. Previous studies have provided inconsistent results on whether G6PD deficient individuals exhibit higher levels of lipid peroxidation than individuals with normal G6PD activity [9,28,29]. Resting levels of protein oxidation as examined by measuring protein carbonyl concentration also did not change at any treatment period. A previous study by Theodorou et al. (2010) [11] showed that protein oxidation levels were similar in G6PD deficient individuals and non-deficient ones. Taken together, G6PD deficient individuals may have developed increased endogenous antioxidant defences in large tissues and do not exhibit higher levels of oxidative stress compared to normal counterparts and ALA supplementation did not provide any additional effect.

Moreover, TBARS and protein carbonyls did not change following acute exercise at any treatment period. These results are not in accordance with the ones from a previous study that has reported increased levels of these indices following acute exercise [9]. It could be hypothesized that muscles of G6PD deficient individuals are not more susceptible to oxidative stress than those of normal counterparts. However, these results could also be attributable to the exercise protocol. Muscle-damaging exercise would provide more information.

## 5. Conclusions

In conclusion, ALA supplementation may contribute to enhanced antioxidant defence in individuals with G6PD deficiency. Nonetheless, ALA supplementation does not change redox responses to acute exercise and does not improve exercise performance. Muscle-damaging eccentric exercise protocols and more time points of blood samples could provide more evidence.

## Figures and Tables

**Table 1:** Physical characteristics of the participants following α-lipoic acid supplementation (ALA) and placebo (PL).

Parameter	ALA		PL	
	Pre	Post	Pre	Post
Body Weight (kg)	81.64 ± 5.65	81.67 ± 5.32	81.53 ± 5.96	82.00 ± 5.86
BMI (kg/m^2^)	25.99 ± 1.50	26.02 ± 1.40	25.94 ± 1.58	26.09 ± 1.53
% Body Fat	25.84 ± 2.16	25.36 ± 2.32	25.43 ± 2.18	26.37 ± 1.92
Waist Circumference (cm)	89.36 ± 8.37	89.14 ± 9.00	88.57 ± 8.78	88.64 ± 8.00
Hip Circumference (cm)	104.7 ± 6.7	104.1 ± 6.7	104.6 ± 6.4	104.3 ± 6.4
WHR	0.853 ± 0.017	0.855 ± 0.018	0.846 ± 0.016	0.849 ± 0.014
Systolic BP (mm Hg)	130.9 ± 11.7	130.0 ± 12.7	130.6 ± 11.1	129.1 ± 12.0
Diastolic BP (mm Hg)	82.0 ± 7.4	80.0 ± 5.9	81.7 ± 6.5	78.6 ± 4.4
Resting HR	63.43 ± 2.57	59.29 ± 1.91 *	62.14 ± 2.53	62.00 ± 2.73
IPAQ (METs/min)	1226 ± 336	1199 ± 281	922 ± 323	981 ± 336

* Significant difference from pre values at the same condition. BMI: Body Mass Index; WHR: Waist to Hip Ratio; BP: Blood Pressure; HR: Heart Rate; IPAQ: International Physical Activity Questionnaire; METs: Metabolic Equivalent of Task.

**Table 2:** Exercise performance parameters following α-lipoic acid supplementation (ALA) and placebo (PL).

Parameter	ALA		PL	
	Pre	Post	Pre	Post
Time to exhaustion (s)	198.9 ± 37.0	246.9 ± 37.6	207.9 ± 37.4	267.4 ± 36.4
MVC (Extensors)				
Left	206.8 ± 18.1	205.3 ± 23.6	196.3 ± 18.8	202.8 ± 22.0
Right	213.8 ± 15.7	231.5 ± 22.5	209.8 ± 10.7	220.5 ± 11.1
MVC (Flexors)				
Left	170.7 ± 14.4	169.3 ± 10.4	164.8 ± 10.9	172.7 ± 12.4
Right	169.2 ± 18.8	169.0 ± 13.8	167.7 ± 14.3	172.2 ± 14.2

MVC: Maximum Voluntary Contraction.
